# Antifungal activity of ETD151 against azole-susceptible and -resistant *Aspergillus fumigatus* clinical isolates

**DOI:** 10.1016/j.crmicr.2025.100486

**Published:** 2025-10-09

**Authors:** Camille Rochard, Jeanne Bigot, Nicolas Millet, Viviane Balloy, Isabel Valsecchi, Françoise Botterel, Romain Morichon, Thierry Fontaine, Loïc Guillot, Philippe Bulet, Christophe Hennequin, Juliette Guitard

**Affiliations:** a Sorbonne Université, Inserm, Centre de Recherche Saint-Antoine, CRSA, F-75012 Paris, France; b AP-HP, Hôpital Saint-Antoine, Service de Parasitologie-Mycologie, Paris, France; c Université Paris-Est Créteil, Ecole nationale vétérinaire d'Alfort, USC ANSES, Dynamyc research team, Créteil, F-94000, France; d Institut Pasteur, Université Paris Cité, INRAE, USC2019, Unité Biologie et Pathogénicité Fongiques, F-75015 Paris, France; e Institut pour l'Avancée des Biosciences (IAB), INSERM 1209, CNRS UMR 5309, Team RNA, Epigenetics and Stress, Université Grenoble Alpes, Grenoble, France; f Plateforme BioPak d’Archamps, Bâtiment Le Forum 2, 218 avenue Marie Curie, 74160 Archamps, France

**Keywords:** Antimicrobial peptides, *Aspergillus fumigatus*, Antifungals, Antimicrobial resistance, Azole resistant strain

## Abstract

Aspergillus fumigatus is an opportunistic pathogen responsible for a wide spectrum of severe diseases, depending on the host's immune status. Current therapeutic options against this fungus are limited, and the emergence of resistance against azole derivatives, which are the first-line therapy, regardless of the clinical form, poses serious clinical challenges. Antimicrobial peptides, now used routinely, are valuable alternatives to conventional antibiotics for treating certain bacterial infections. In this study, we investigated the antifungal potential of ETD151, a synthetic peptide, against *A. fumigatus* clinical strains. Using cell-free *in vitro* assays and primary human bronchial epithelial cell (PHBEC) infection models, we demonstrated that ETD151 significantly reduces A. fumigatus hyphal growth by 89 % and metabolic activity by 70 %. This was associated with marked morphological alterations that occurred both in azole-susceptible and azole-resistant strains. Rescue assay and analysis of a glucosylceramide-deficient strain revealed that those sphingolipids are a molecular target of ETD151 in *A. fumigatus*. Importantly, ETD151 showed no cytotoxicity toward PHBECs and maintained its antifungal activity in co-culture conditions. These findings support the potential of ETD151 as a promising antifungal candidate for the treatment of A. fumigatus-associated diseases.

## Highlights

• ETD151 is active against azole sensitive or resistant *A. fumigatus* clinical strains.
• ETD151 targets glucosylceramides of the fungal membrane.
• *A. fumigatus* does not develop resistance to ETD151.
• ETD151 remains active in a model of *A. fumigatus* infected-bronchial epithelial cells.

## Graphical abstract

**Figure:**
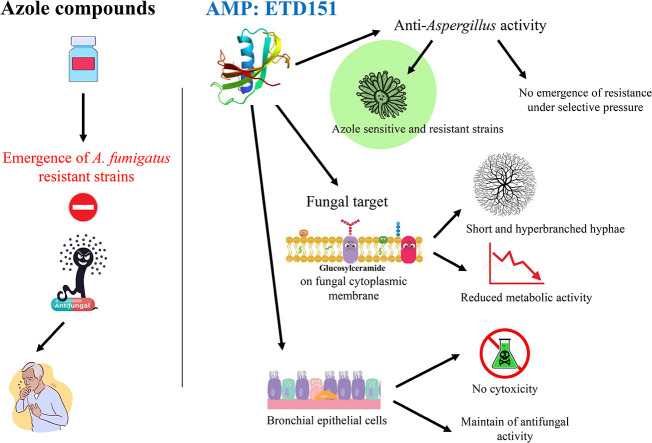

## 1 Introduction

Over the last decades, antimicrobial resistance has become a major global health crisis. (Denning, 2024). While much of the focus has been on bacteria, antifungal resistance is a rising yet underrecognized and underestimated part of this crisis (Van Rhijn et al., 2024). This includes both intrinsically resistant species, such as *Candida auris* and *Trichophyton indotineae* (Desoubeaux et al., 2022; Jabet et al., 2023)*,* as well as species that have acquired resistance, notably azole-resistant strains of *Candida parapsilosis* and *Aspergillus fumigatus* (McHugh et al., 2025*;* Gong et al., 2024). In light of its rising resistance and substantial clinical impact, *A. fumigatus* was recently classified by the World Health Organization as a critical fungal pathogen. In doing so, the WHO called for improved surveillance, optimized use of existing antifungal drugs, and the development of novel therapeutic strategies (Fisher and Denning, 2023).

*A. fumigatus* is a ubiquitous environmental mould whose airborne conidia are inhaled daily. In most individuals, this exposure is harmless due to efficient immune and mucociliary defenses. However, depending on the host’s immune status and underlying lung conditions, inhalation can lead to a spectrum of diseases. These range from allergic responses, such as severe asthma or allergic bronchopulmonary aspergillosis (ABPA), to invasive infections (IA) in immunocompromised patients (Latgé and Chamilos, 2019). In between, *A. fumigatus* can chronically colonize the lungs of individuals with impaired mucociliary clearance, such as patients with cystic fibrosis (CF), chronic obstructive pulmonary disease (COPD), or chronic asthma, potentially leading to chronic pulmonary aspergillosis (CPA).

Currently, the therapeutic arsenal against *Aspergillus* spp. remains limited. Among available antifungals, new-generation azole derivatives (voriconazole, posaconazole, isavuconazole) are the first-line treatment for aspergillosis due to their intrinsic antifungal activity, safety profile, and oral bioavailability (Patterson et al., 2016; Denning, 2016). However, the emergence of azole-resistant strains since the 2000s represents a growing threat to effective therapy (Snelders et al., 2008). This phenomenon is mostly attributed to the widespread use of azole-based fungicides in agriculture, although long-term azole therapy in patients with ABPA or CPA also contributes to selective pressure (Morio et al., 2012; Takeda et al., 2022). Resistance in *A. fumigatus* typically involves mutations in the *cyp51A* gene, which encodes the azole target enzyme C14-alpha-demethylase essential for ergosterol biosynthesis (Mellado et al., 2001; Wiederhold and Patterson, 2015). Consequently, azole-resistant *A. fumigatus* strains are more prevalent in patients with ABPA or CPA. Prevalence varies geographically, with rates of 13 % and 25 % reported among CPA patients in India and Japan, respectively (Singh et al., 2020; He et al., 2024). In France, the prevalence among CF patients reaches 8 % (Morio et al., 2012).

In this context, antimicrobial peptides (AMPs) have emerged as a promising alternative to conventional antibiotics (Rima et al., 2021). AMPs are short polypeptides of 12 to 50 amino acids, synthesized by virtually all living organisms as part of the innate immune defense (Bahar and Ren, 2013). Physicochemical properties, such as charge, size, hydrophobicity, amphiphilicity, or secondary structure, influence the spectrum of activity of AMPs (Browne et al., 2020; Hong et al., 2024; Krupińska and Bogucki, 2024). Their primary mechanism of action involves direct disruption of microbial membranes, resulting in increased membrane permeability, leakage of cytoplasmic contents and cell death. Other mechanisms, such as inhibition of nucleic acid synthesis and cytosolic enzyme activities, have also been described (Haney et al., 2017). Although some bacterial resistance has been observed, it remains uncommon, likely because these peptides target conserved membrane components difficult to alter without compromising cell viability (Bahar and Ren, 2013; Cassone et al., 2009). Interestingly, synergistic effects of combinations of AMPs with conventional antibiotics have been demonstrated (Dos Reis et al., 2023). Some AMPs, such as daptomycin and colistin, are already used routinely (Boparai and Sharma, 2020). Daptomycin, which disrupts the cytoplasmic membrane of Gram-positive bacteria such as Staphylococcus aureus, is approved mainly for complicated skin and soft tissue infections, S. aureus bacteremia (MRSA), and right-sided endocarditis (Hair et Keam, 2007). Importantly, colistin is used against multidrug-resistant Gram-negative infections, including complicated urinary tract infections (Abu-Farha et al., 2025). In addition to their antimicrobial effects, certain AMPs exhibit immunomodulatory properties by stimulating the production of pro- or anti-inflammatory cytokines. For example, human defensins and cationic peptides can modulate cytokine expression (IL-6, TNF-α) and influence immune cell activity (Scott et al., 2002; Hancock, 2001; Lee et al., 2022), highlighting their dual role in host defense and immune regulation.

ETD151, a 44-amino-acid recombinant amphiphilic AMP, was engineered from a consensus sequence shared by the insect-derived peptides, heliomicin and ARD1 originally isolated from *Heliothis virescens* and *Archaeoprepona demophon,* respectively (Thevissen et al., 2004; Larwood et al., 2020; Helmerhorst et al., 2001). Previous studies showed that ETD151 inhibits the growth of *Botrytis cinerea,* an important phytopathogen fungus. ETD151 caused hyperbranched and hyphal swelling *in vitro* through a mechanism of action thought to involve membrane targeting (Aumer et al., 2020; Kharrat et al., 2025).

**Fig. 1:**
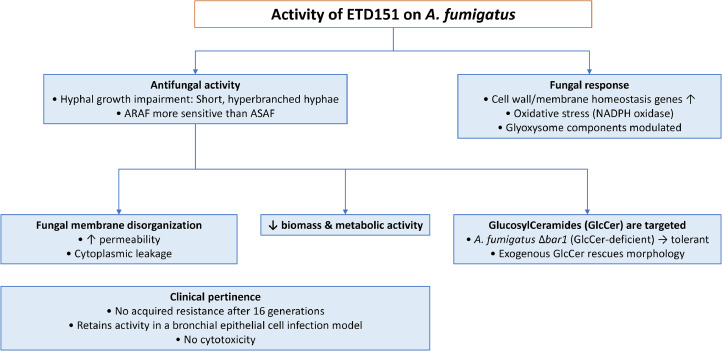
Flow chart summarizing the observed antifungal effect of ETD151 on A. fumigatus.

In the present study, we assessed the anti-*Aspergillus* activity of ETD151 against both azole-susceptible (ASAF) and azole-resistant (ARAF) *A. fumigatus* clinical isolates. We also employed an *in vitro* infection model using primary human bronchial epithelial cells (PHBECs) to assess its effects on fungal clearance and host immune responses. Our findings highlight ETD151’s potential as a membrane-active peptide that targets *A. fumigatus* glucosylceramides (GlcCer), retains efficacy in an epithelial environment, and exhibits no detectable cytotoxicity. These results support its potential as a therapeutic candidate against azole-susceptible and azole-resistant aspergillosis (Fig. 1).

## 2 Materials and methods

### 2.1 Reagents

ETD151, obtained from Bulet-EIRL (Archamps, Grenoble), was solubilized in sterile distilled water and stored at 4 °C. Amphotericin B, caspofungin, itraconazole, and voriconazole were purchased from Sigma-Aldrich (USA), SYTOX Green and Resazurin from Thermofisher (France), and Soy derived- GlcCer from Avanti Polar Lipids (USA). Yeast Peptone Dextrose broth (YPD) was purchased from Sigma (USA). Resazurin was solubilized in distilled water, and amphotericin B (50 mg/mL), caspofungin (5 mg/mL), voriconazole (3.2 mg/mL), and itraconazole (10 mg/mL) in DMSO. GlcCer were solubilized in ethanol 100 % (10 mg/mL). All reagents were adjusted to required concentrations in culture medium.

For confocal microscopy analysis, primary antibodies included mouse-anti-claudin (AB374900) and rabbit-anti-α-tubulin (AB52866), both from Abcam (United Kingdom). Secondary antibodies were Alexa Fluor 647 conjugated anti-mouse (A11012) and Alexa Fluor 594-conjugated anti-rabbit (A21237) supplied by Invitrogen (USA). ProLong from Thermofisher (France) was used for mounting samples.

### 2.2 *A. fumigatus* strains, culture and preparation of inoculum

Four ASAF clinical strains, including two reference strains (CEA10 and Af293), and four ARAF clinical strains were used in this study (Table 1). Furthermore, an *A. fumigatus Δbar1* strain was generated using homologous recombination techniques from *A. fumigatus* CEA10 *Δku80* and GlcCer loss of production was tested using thin layer chromatography (TLC), as described in the Supplementary Data (experimental method 1.1). Chromatographic profiles of the *A. fumigatus* parental as well as the Δ*bar1* mutant strain were analyzed, as described in the Supplementary Data (experimental method 1.1).

**Table 1:** Genotypic and phenotypic characteristics of strains used in this study. MICs were determined using the EUCAST methodology for molds version 9.4. Susceptibility categorizations were assigned based on the clinical breakpoints established by EUCAST version 11.0.

Strains		*Cyp51* genotype	ITZ		VRZ		PSZ		Clinical origin
Category	name		MIC (mg/L)	S/ATU/R	MIC (mg/L)	S/ATU/R	MIC (mg/L)	S/ATU/R	
ASAF	S1	WT	0.5	S	0.19	S	0.094	S	CPA
	S2	WT	0.5	S	0.25	S	0.094	S	CF
	CEA10 =S3	WT	0.5	S	0.19	S	0.094	S	IA
	Af293 = S4	WT	1.5	ATU	0.75	S	0.25	ATU	IA
ARAF	R1	TR34/L98H	32	R	4	R	0.5	R	CF
	R2	TR34/L98H	32	R	24	R	0.5	R	
	R3	P216L	16	R	0.125	S	0.75	R	
	R4	G54W	32	R	0.19	S	32	R	

ARAF: azole- resistant *A. fumigatus* strains, ASAF: azole-susceptible *A. fumigatus* strains, ATU: Area of Technical Uncertainty, CF: Cystic fibrosis, CPA: chronic pulmonary aspergillosis, EUCAST: European Committee on Antimicrobial Susceptibility Testing, IA: Invasive aspergillosis, ITZ: itraconazole, MIC: minimum inhibitory concentrations, PSZ: Posaconazole, R: resistant, S: susceptible, VRZ: voriconazole.

For inoculum preparation, strains were cultured on Sabouraud Dextrose Agar slants (Biomerieux, France) at 37 °C for 7 days. Conidia were harvested in phosphate-buffered saline (PBS) with 0.1 % Tween 20, filtered through a 40-μm nylon mesh to remove hyphal, and stored at 4 °C before use. Conidia were diluted to the desired concentration in Sabouraud broth, immediately before use.

### 2.3 Determination of *A. fumigatus* ETD151-induced growth alterations

The impact of ETD151 on *A. fumigatus* morphology was determined by incubating *A. fumigatus* conidia (5 × 10^4^ conidia/mL) in Sabouraud broth on 8-well chamber slides (Lab-Tek, ThermoScientific®) for 15 h at 37 °C with ETD151 (5 µM). The impact of ETD151 was also determined on preformed hyphae: conidia were incubated for 15 h at 37 °C to obtain hyphae, and then treated with ETD151 (10 µM) for an additional 15 h at 37 °C. Hyphal morphology was examined by optical microscopy (200x magnification, Olympus CKX53) and hyphal lengths measured using ImageJ software.

Fungal biomass was quantified using a Crystal Violet assay. A suspension of 10⁵ conidia/mL was incubated in Sabouraud broth for 20 h at 37 °C with 5 µM ETD151. After washing, hyphae were stained with 130 µL of 0.01 % Crystal Violet (incubation at room temperature for 20 min), rinsed with distilled water, air-dried overnight, destained with 130 µL of 30 % acetic acid (shaking at 250 rpm for 20 min), and absorbance measured at 560 nm.

Minimal Effective Concentration (MEC) was determined using an EUCAST modified method (Microsoft Word - EUCAST EDef 9.4 method for susceptibility testing of moulds.docx): RPMI 1640 medium was supplemented with 1 % Hepes. *A. fumigatus* inoculum (10^4^ conidia/mL in 0.1 % Tween 20) was added to sterile 96-well flat-bottom microtiter plates (Greiner bio one®, Germany) containing 100 µL of two-fold serial dilutions of ETD151 (ranging from 0.0097 to 2.5 µM). Controls included wells with medium only, wells without ETD151, and wells containing itraconazole at 1 and 8 mg/L as reference antifungal controls. Triplicates were incubated 24 h at 37 °C in a humidified chamber. MEC was determined microscopically as the lowest concentration of ETD151 inducing a morphological changes (Siopi et al., 2023; Lockhart et al., 2011).

### 2.4 Measure of *A. fumigatus* metabolic activity

Metabolic activity was quantified using a resazurin-based assay. Metabolically active cells reduce resazurin to resofurin, whose fluorescence is measured at 570 nm using a spectrofluorometer. In black 96-well microplates with transparent bottoms (Greiner bio one®, Germany, 655,090), 10^4^ *A. fumigatus* conidia were inoculated into 90 µL of Sabouraud broth containing 10 µL of antifungal molecules (ETD151 (0–10 µM), or itraconazole (0.5 and 8 mg/L) or AMB (1 mg/L)), and each well were filled with 100 µL of resazurin (0.001 %). After a 15-hour incubation time at 37 °C, fluorescence was measured at 570 nm using a Fluostar OPTIMA spectrofluorometer (BMG labtech). Metabolic activity was expressed as a percentage relative to untreated controls. Amphotericin B was used as a positive control in all metabolic activity assays.

### 2.5 Measure of *A. fumigatus* membrane permeabilization

To assess membrane integrity, 200 µl of a 2.5 × 10^4^/mL *A. fumigatus* conidia suspension were incubated with ETD151 for 15 h at 37 °C. Hyphae were stained with 1 % Calcofluor White (Sigma-Aldrich, USA), to visualize chitin-containing cell walls, 2.5 µM SYTOX Green (Thermofisher, France), a nucleic acid dye excluded by intact membranes to detect permeabilized cells. Samples were examined by fluorescence microscopy (Olympus CKX53) using dual-wavelength excitation (365–405 nm for Calcofluor White and 490–510 nm for SYTOX Green). Membrane permeabilization was quantified as the proportion of SYTOX Green-positive hyphae to the total number of Calcofluor White-stained hyphae.

### 2.6 Assessment of ETD151-induced selective pressure

To evaluate the potential for resistance development against ETD151, *A. fumigatus* conidia (50 to 250 conidia/mL) were first grown in the presence of 1.25 µM ETD151 for 24 h in liquid Sabouraud medium. The resulting mycelium was then transferred to malt agar plates and incubated at 37 °C for 48 h. Conidia harvested from these cultures were used to repeat the procedure for a total of 16 consecutive passages. The ETD151 MEC and metabolic activity of the passaged strains were subsequently assessed and compared with those of the parental strain to detect any changes in susceptibility.

### 2.7 Effect of ETD151 in combination with conventional antifungal agents

Resazurin assay was used to determine the MA_50_, defined as the concentration of drug that inhibits metabolic activity by 50 %, of voriconazole, caspofungin and ETD151 toward each strain. We first determined the MA_50_ of each strain for the antifungal molecules tested (voriconazole, caspofungin, and ETD151). Then, we determined the metabolic activity of each strain when voriconazole or caspofungin was combined with ETD151, used at the MA_50_ previoulsy determined. 100 µl of a 10^5^ conidia/mL suspension were incubated in Sabouraud broth with caspofungin or voriconazole in combination with ETD151, added simultaneously at their respective MA_50_ concentrations. After adding 100 µL of resazurin (0.001 %), plates were incubated at 37 °C for 15 h. Comparisons were made between the MA_50_ values of individual drugs and their combination with ETD151.

### 2.8 Transcriptomic analysis

RNA sequencing (RNA-seq) was performed to analyze the transcriptomic response of A. fumigatus to ETD151. Conidia of the Af293 reference strain were inoculated into flasks (TPP® 90,026 tissue culture flask 25) containing 10 mL of YPD (10^6^ conidia/mL) and incubated for 15 h at 37 °C under agitation at 100 rpm, and further treated or not with ETD151 (0.156 µM) for 30 min, 1 hour or 6 h. mRNA was then extracted using the Nucleospin miRNA kit (Macherey Nagel®), as described in the Supplementay data (Experimental method 1.2). The raw sequencing data, including details on data processing, alignment, and differential gene expression analysis, are provided in the Supplementary data (Experimental method 1.2).

### 2.9 Antifungal activity of ETD151 on *A. fumigatus-*infected primary human bronchial epithelial cells

Primary human bronchial epithelial cells (PHBECs) (Epithelix, Plan-les-Ouates, Switzerland) from three healthy donors: (donor 1: batch MD0889, age: 17, sex: male; donor 2: batch MD0786, age: 64, sex: female, donor 3: batch MD0937, age: 29, sex: male, certified mycoplasma-free were cultured in complete hAEC medium (Epithelix) and grown in an air-liquid interface (ALI). Epithelial integrity was assessed by trans-epithelial electrical resistance (TEER) measurement three days prior infection, and after 15 h of increasing concentrations of ETD151. Toxicity was expressed as TEER (Measured resistance - Blank resistance) x insert membrane area.

For infection, 0.5 × 10^6^ *A. fumigatus* ATCC13073 GFP-expressing conidia were applied on the apical surface of PHBECs (Multiplicity of Infection (MOI 1)) and incubated with or without ETD151 (5 µM) for 15, 24 and 48 h at 37 °C. Inflammatory response was quantified after 15 h of ETD151 treatment by measuring IL-6 and IL-8 by ELISA (R&D System, USA) in apical and basal supernatants using 3.3′5.5′-tetramethylbenzidine (Cell Signaling Technology, Danvers, MA, USA) and FLUOstar OPTIMA plate reader (BMG Labtech, Champigny-sur-Marne, France) with MARS Data Analysis Software v3.01 R2 (BMG Labtech).

Hyphal morphology was examined by confocal microscopy, after fixation in 4 % paraformaldehyde (15 min), and blocking with PBS plus 5 % bovine serum albumin (1 hour at room temperature). Cells were stained with anti-claudin and anti-α tubulin antibody (1:200) for 3 hours followed by appropriate secondary antibodies (1:2000). After final washes (three times with PBS and once with distilled water), slides were mounted with ProLong medium. Confocal fluorescence imaging was performed using an Olympus FV3000 microscope (x400) and images were processed with the Imaris software V9.9 (Oxford Instruments, Abingdon-on-Thames, UK) (Ames, 1966).

Antifungal activity was assessed by lysing the PHBEC, after 15 h of ETD151 treatment, to recover endocytosed and adherent conidia, plating the lysates on malt agar, and incubating for 24 h at 37 °C. Colony Forming Units (CFU) were enumerated, and results were expressed as percentages relative to the untreated control.

### 2.10 Statistical analysis

Statistical analyses were performed using GraphPad Prism version 10 software. Groups were compared using the Student’s *t*-test (two groups) or multiple comparisons were made using One-way ANOVA test with Bonferroni correction (> two groups). Data were presented as mean ± standard deviation (SD).

## 3 Results

### 3.1 ETD151 impacts ASAF and ARAF growth

**Fig. 2:**
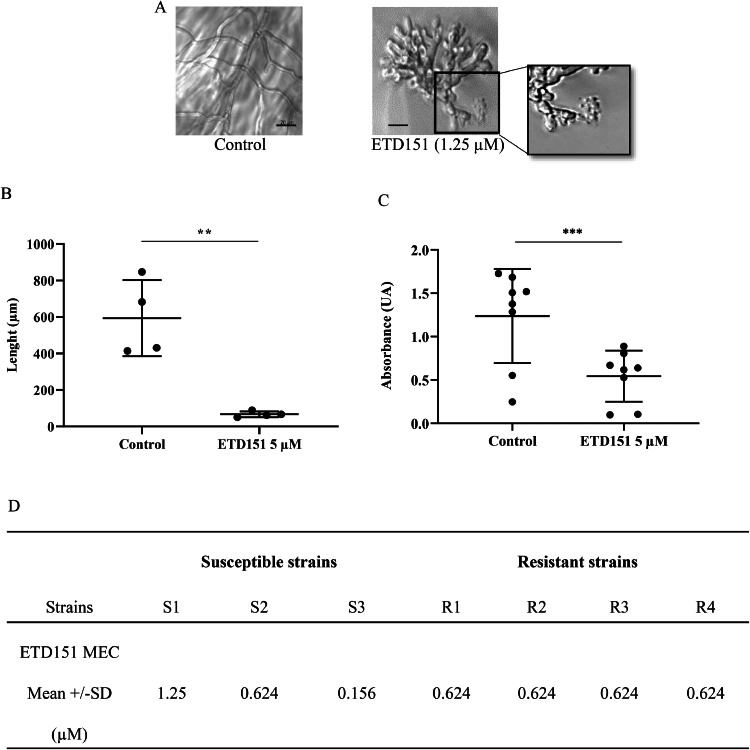
(A) Representative microscopic images of ASAF S1 strain after 15 h incubation, either untreated (left) or treated (right) with 1.25 µM ETD151 (scale bar = 20 µM). (B) Hyphal length measurement of Aspergillus after treatment with 5 µM ETD151 for 15 h (n = 4). Data represent mean ± SD of the hyphal length of four tested strains. (C) Fungal biomass quantification in the presence of 5 µM ETD151 after 20 h of incubation. Data represent mean ± SD of eight tested strains, each point represents a technical triplicate. (D) Determination of Minimum Effective Concentrations (MECs) of ETD151 against ASAF and ARAF clinical isolates. Data represent mean ± SD with three biological replicates per strain for each of the three ASAF and the four ARAF tested strains. Statistical analysis was performed using an unpaired *t*-test (**p < 0.001).

We first assessed the impact of ETD151 on *A. fumigatus* morphology across a panel of clinical isolates (Table 1), including four ASAF (S1, S2, S3 and S4) and four ARAF (R1, R2, R3, R4) strains. Conidia were incubated in the presence of ETD151 for 15 h at 37 °C in Sabouraud broth to allow germination and hyphal development. Microscopic examination of treated hyphae exhibited striking morphological alterations, including short, stubby, and highly branched hyphal clusters in contrast to the untreated control, which displayed typical long, thin, and extended hyphae (Figure 2A; movie S1). This aberrant morphology was accompanied by cytoplasmic leakage at hyphal tips (inset Fig. 2A and movie S1). This morphology was also observed when preformed hyphae were incubated for 15 h with ETD151 (movie S2). Length of *A. fumigatus* hyphae decreased significantly from 594 µm ± 209 µM to 67 µM ± 16 µm when treated with 5 µM ETD151 (Fig. 2B). In the same way, biomass was reduced by 42 % after treatment with ETD151 (from 1.2 ± 0.5 arbitrary unit (AU) to 0.5 ± 0.3 AU when treated with ETD151), using crystal violet measurement (Fig. 2C).

Taking as a model the strategy to assess the *in vitro* efficacy of echinocandins, we determined the MEC of ETD151 using a modified version of the EUCAST microdilution method. MEC determination was chosen because no complete growth inhibition was observed in standard MIC assays. MECs for ASAF and ARAF strains were in the same range: 0.156 to 1.25 µM, with a mean of 0.677 ± 0.549 µM, *versus* 0.624 µM (Figure 2D; Figure S5).

### 3.2 ETD151 reduces the metabolism of azole-susceptible and -resistant *A. fumigatus*

**Fig. 3:**
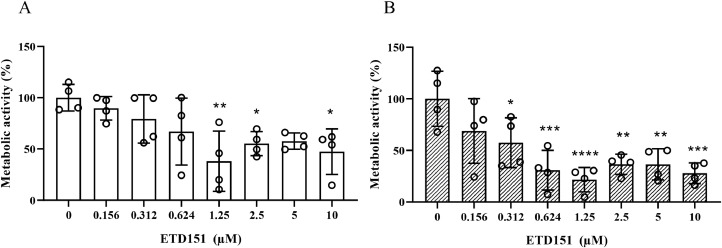
Effect of ETD151 on metabolic activity was measured by resazurin assay after 15 h of incubation at 37 °C in Sabouraud medium (A-ASAF strains; B-ARAF strains). Metabolic activity was expressed as a percentage relative to untreated controls. Data are expressed as mean ± SD of metabolic activity obtained for the four ASAF and four ARAF tested strains. Each point represents a technical replicate. Statistical significance between treated and untreated conditions was determined using one-way ANOVA with Bonferroni’s multiple comparisons test. *p < 0.0001.

The impact of ETD151 and amphotericin B, used as a control, on the metabolic activity of ASAF and ARAF strains was measured using a resazurin assay. As expected, 15 h treatment with amphotericin B (1 mg/L) reduced *A. fumigatus* metabolic activity by 86 ± 5 % (*p* < 0.0001) for ASAF and ARAF strains, while itraconazole (0.5 mg/L) only reduced ASAF metabolic activity by 92.2 ± 2.5 % (p < 0.0001). ETD151 treatment led to a dose-dependent reduction in metabolic activity up to 1.25 µM (Fig. 3). At this optimum concentration, mean metabolic activity decreased by 62 ± 29.4 % in ASAF strains (*p* *=* 0.0002) and by 78.4 ± 11.8 % in ARAF strains (*p* = 0.0004), relative to their respective untreated control group. However, it is important to note that these reductions, while substantial, do not represent complete inhibition of A. fumigatus metabolic activity.

### 3.3 ETD151 induces membrane permeabilization in *A. fumigatus* hyphae

**Fig. 4:**
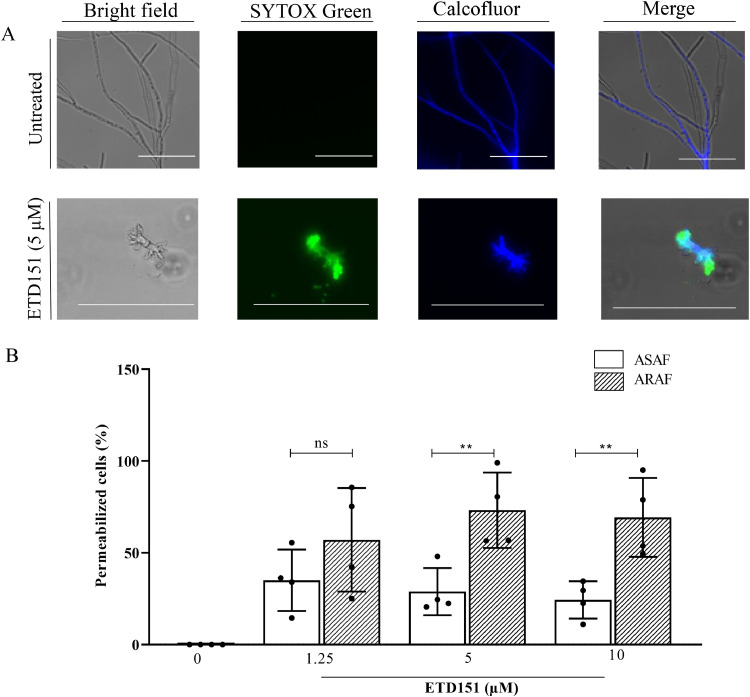
(A) Representative bright-field and fluorescence images (x20) of hyphae treated for 15 h with 5 µM ETD151. Images were acquired using an Olympus FV3000 microscope. Scale bar = 150 µm. (B) Quantification of Sytox Green-positive ASAF (white bars) and ARAF (hatched bars) hyphae after 15 h of incubation with 0, 1.25, 5, 10 μM of ETD151. Data are expressed as mean ± SD of the percentage of permeabilized cells for four ASAF and four ARAF tested strains. Each point represents two technical replicates. Statistical significance between ASAF and ARAF strains treated with ETD151 was determined using one-way ANOVA with Bonferroni’s multiple comparisons test. **p.

The membrane permeabilization of *A. fumigatus* hyphae following ETD151 treatment was evaluated using Sytox Green and Calcofluor White staining (Fig. 4A). In untreated controls, no Sytox Green is staining, indicating the integrity of the membranes. In contrast, exposure to ETD151 led to detectable Sytox Green uptake starting at 1.25 µM, consistent with altered membrane integrity (Fig. 4B). Notably, at 5 and 10 µM ETD151, ARAF strains showed a significantly higher proportion of permeabilized hyphal (42.5 ± 21 %) compared to ASAF strains (27.5 ± 13 %), suggesting greater susceptibility of resistant isolates.

### 3.4 ETD151 maintains its efficacy after serial rounds of exposure

**Fig. 5:**
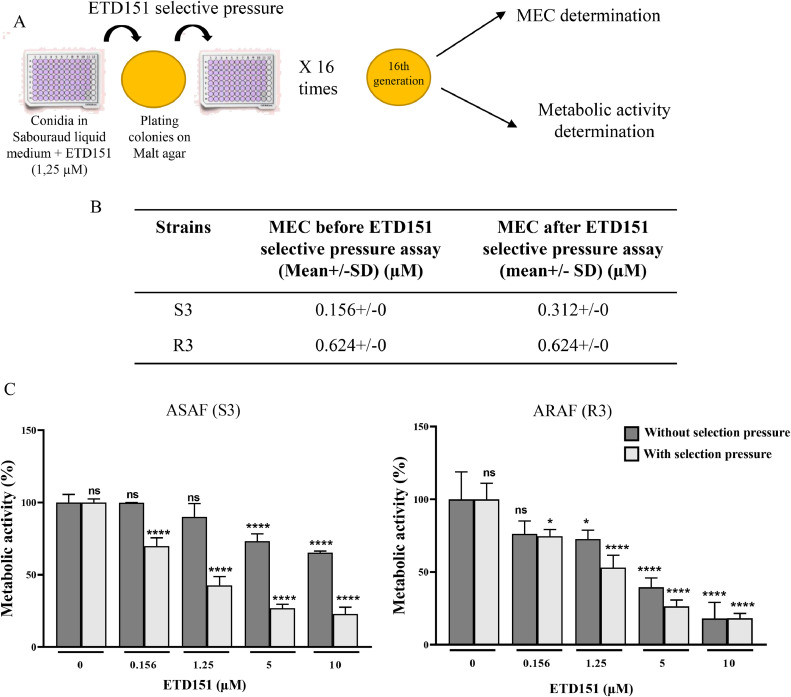
(A) Experimental design of the ETD151 selective pressure assay. (B) MEC values for the parental and the derived A. fumigatus strains treated with ETD151. Values represent the mean of three independent experiments (mean ± SD). (C) Metabolic activity of the parental and the derived A. fumigatus strains treated with ETD151. Histograms represent the percentage of metabolic activity of parental strains (light gray) and the derived A. fumigatus strains treated with ETD151 (light gray). Data are expressed as the mean of three biological replicates of each tested strains. Statistical significance between untreated parental strain and derived A. fumigatus strains treated with ETD151 was determined using one-way ANOVA with Bonferroni’s multiple comparisons test (n = 3). *p < 0.0001.

To evaluate whether repeated exposure to ETD151 leads to the emergence of resistance, S3 and R3 strains were subjected to 16 successive rounds of treatment with 1.25 µM ETD151 (Fig. 5A). We found that the MEC of the derived isolates remained unchanged (less than one 2-fold dilution) compared to their respective unexposed parental strain (Fig. 5B). Similarly, the metabolic activity inhibition was preserved in the derived isolates and was even significantly stronger in ASAF S3 (Fig. 5C). These findings suggest that *A. fumigatus* did not develop resistance to ETD151 under our experimental conditions.

### 3.5 ETD151 has an additive effect with caspofungin

**Fig. 6:**
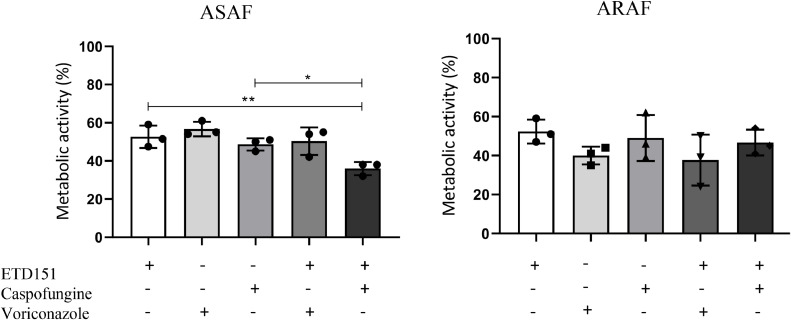
Analysis of additive effect of ETD151 with caspofungin or voriconazole on the inhibition of the metabolic activity of ASAF and ARAF strains. Metabolic activity was expressed as a percentage relative to untreated controls. Data are expressed as mean ± SD of metabolic activity obtained for the three ASAF and three ARAF tested strains. Statistical significance between treated and untreated conditions was determined using one-way ANOVA with Bonferroni’s multiple comparisons test. *p < 0.005.

To determine whether ETD151 acts independently of conventional antifungal agents, we investigated its efficacy in combination with caspofungin or voriconazole against ASAF and ARAF strains. We defined MA_50_ of voriconazole, caspofungin and ETD151 for each strain (Table S1). For each strain, combination of drugs at their MA_50_ concentration were used to determine MA_50_ values of the combination. The results showed that only the combined treatment, ETD151 + caspofungin has additive effect on ASAF strains compared to monotherapy with ETD151 (p < 0.005) or caspofungin (p < 0.05). No effect was observed with voriconazole combination on ASAF strains, nor with ARAF strains (Fig. 6).

### 3.6 ETD151 targets glucosylceramides in *A. fumigatus*

**Fig. 7:**
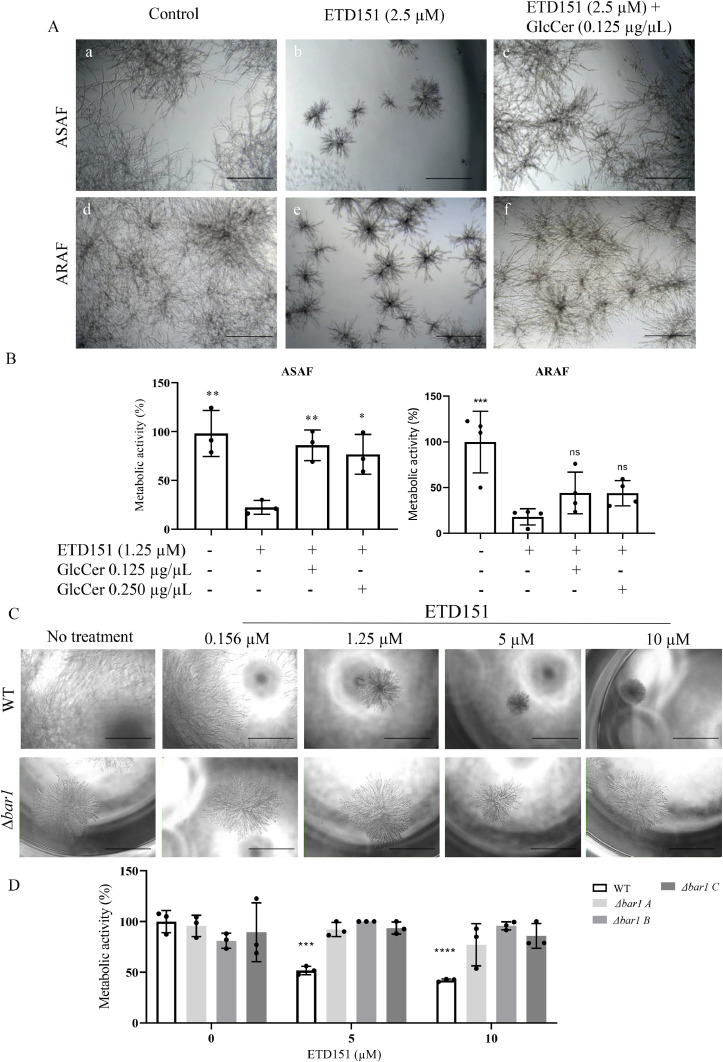
(A) Representative microscopic image of hyphal morphology of one ASAF and one ARAF strain treated (b, e) or not (a, d) with 2.5 µM ETD151, or treated (e, f) with 2.5 µM ETD151 plus 0.125 µg/µL soyderived GlcCer (B) Metabolic activity of ASAF and ARAF strains following ETD151 treatment with or without GlcCer measured by resazurin assay. Results are expressed as percentages relative to untreated controls. Data represent the mean of metabolic activity of three ASAF and four ARAF tested strains. Each point represents a technical duplicate. Statistical significance between strains treated only with ETD151 and strains untreated or treated with ETD151 and GlcCer supplementation was determined using one-way ANOVA with Bonferroni’s multiple comparisons test. (C) Representative bright-field images of ∆ku80 (WT) or ∆bar1 hyphae after exposure to increasing concentrations of ETD151 (0.156–10 µM) for 15 h. Scale bar = 300 µm. (D) Metabolic activity of ∆ku80 and ∆bar1 strains after exposure to increasing concentrations of ETD151 (0–10 µM) for 15 h. Each histogram represents the mean ± SD of metabolic activity of three independent experiments performed for each strain, normalized to the untreated ∆ku80 control. Each point represents a technical duplicate. Statistical significance between untreated parental strain and derived A. fumigatus strains treated with ETD151 was determined using one-way ANOVA with Bonferroni’s multiple comparisons test. *p < 0.05, **p < 0.005, ***p < 0.001, ****p < 0.0001.

Based on previous findings, (Aumer et al., 2020; Kharrat et al., 2025) we investigated whether GlcCer function as a target of ETD151 in *A. fumigatus.* Growth medium supplementation with soy-derived GlcCer visually restored hyphal morphology in both ASAF and ARAF strains treated with 1.25 µM ETD151 (Fig. 7A). GlcCer at 0.125 μg/μL also rescued significantly the metabolic activity of ASAF strains, reaching 86±16 % of the control. In contrast, ARAF strains did not recover their metabolic activity, even at the highest GlcCer concentration tested (0.25 μg/μL) (Fig. 7B).

To further confirm GlcCer as a direct target of ETD151 in *A. fumigatus*, we generated a Δ*ku80* strain deleted for the *bar1* gene. *bar1* encodes a ceramide synthase that catalyzes the N-acylation of sphingosine with a fatty acid to form ceramide, the direct precursor of GlcCer. Thin-layer chromatography confirmed the absence of GlcCer in the resulting *∆bar1* strains (strains A and B) (Figure S2). Although the *∆bar1* strains were viable at all tested temperatures (25 °C to 42 °C), they exhibited reduced colony diameters compared to the parental *∆ku80* strain (Figure S3). In addition, ∆*bar1* strains displayed inherently hyperbranched and shortened hyphae, mimicking the phenotype observed after ETD151 treatment of the wild-type strain (Fig. 7C). No additional morphological alteration was observed when *Δbar1* strains were treated with ETD151 (Fig. 7C). Upon exposure to ETD151 (5 or 10 µM), the metabolic activity of *∆bar1* strains remained unaffected, whereas *∆ku80* strain showed a significantly reduced metabolic activity (49 %) (Fig. 7D). Altogether, these findings suggest that GlcCer are the main target of ETD151 and underscore its role in maintaining normal hyphal architecture.

### 3.7 ETD151 triggers a fungal stress response and disrupts cell wall homeostasis

To further explore the broader fungal response to ETD151, we performed a time-course transcriptomic analysis on hyphae of the *A. fumigatus* reference strain Af293, whose genome has already been sequenced, following exposure to 0.156 µM ETD151 for 30 min, 1 hour, and 6 h. Significant transcriptional changes (p < 0.05) were detected at all-time points compared to untreated controls, with 90 genes modulated at 30 min (47 upregulated, 43 downregulated), 25 genes at 1 hour (23 up, 2 down), and a marked increase to 821 differentially expressed genes at 6 h (596 up, 225 down).

**Fig. 8:**
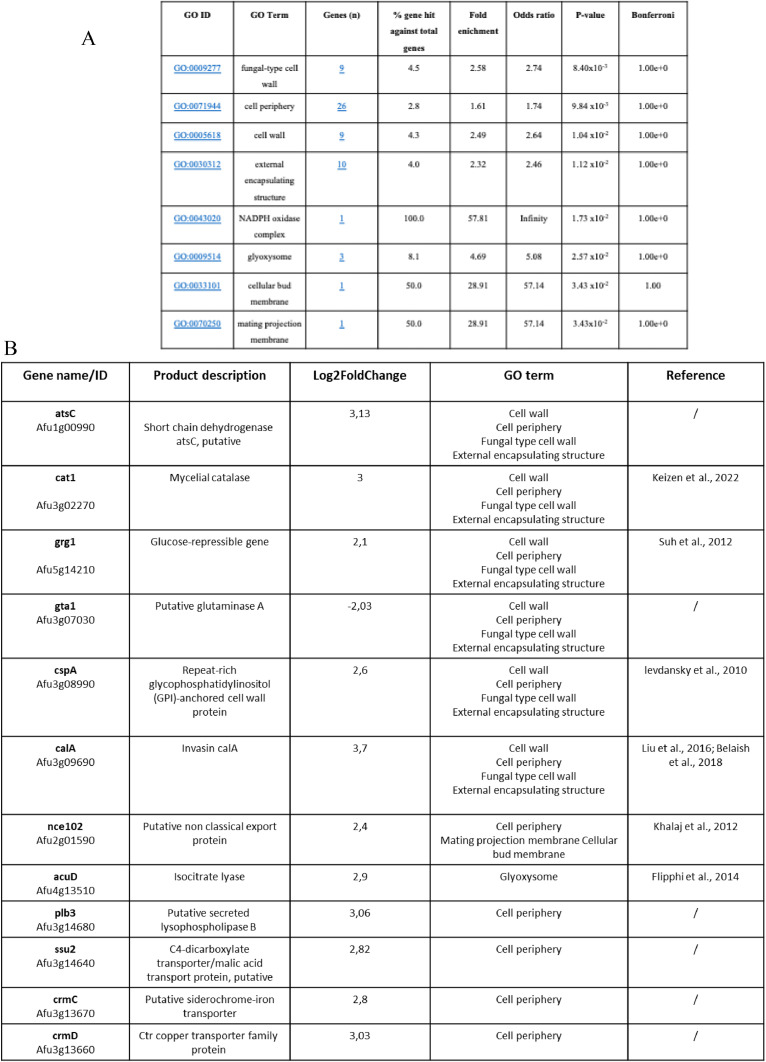
Gene Ontology enrichment of differentially expressed genes in ETD151-treated A. fumigatus Af293 hyphae. (A) Enrichment analysis of significantly regulated GO terms in differentially expressed genes of A. fumigatus Af293 following 6 h of ETD151 treatment. The table highlights the cellular components most affected by the treatment, providing insights into the functional pathways and cellular mechanisms altered in response to ETD151. (B) List of the differentially expressed genes in A. fumigatus Af293 after ETD151 treatment, classified through Gene Ontology cellular component and annotated.

To interpret these changes, a functional enrichment analysis was conducted on the 171 annotated genes (161 genes upregulated and 10 downregulated) that showed a log₂ fold change greater than 2 or less than –2 at 6 h. FungiDB database was used to classify genes according to Gene-Ontology (GO) cellular component. This analysis identified eight families, six of which were related to fungal membrane or cell wall homeostasis: fungal-type cell wall, cell wall, cell periphery, cell bud membrane, mating projection membrane, and external encapsulating structure (Fig. 8A). Among genes belonging to these families, we found *cat1, grg1, CspA, CalA, nce102, and acuD*, upregulated, and *gta1* downregulated. All the changes in the expression of these genes line up with the unusual shapes and structures observed after ETD151 treatment, giving a clue about how it disrupts the cell wall and membrane.

Among the upregulated genes, we found one gene (AFU6G13350) belonging to the “NADPH oxidase complex” described to have superoxide-generating NADPH oxidase activity. NADPH oxidase is a key enzyme involved in fungal respiration, oxidative stress response, and virulence (Kim, 2014)*.* Additionally, several differentially expressed genes were found in the glyoxysome family, glyoxysome being an organelle implicated in stress adaptation (De Castro et al., 2023) (Fig. 8B).

### 3.8 ETD151 retains its antifungal activity in an epithelial infection model

**Fig. 9:**
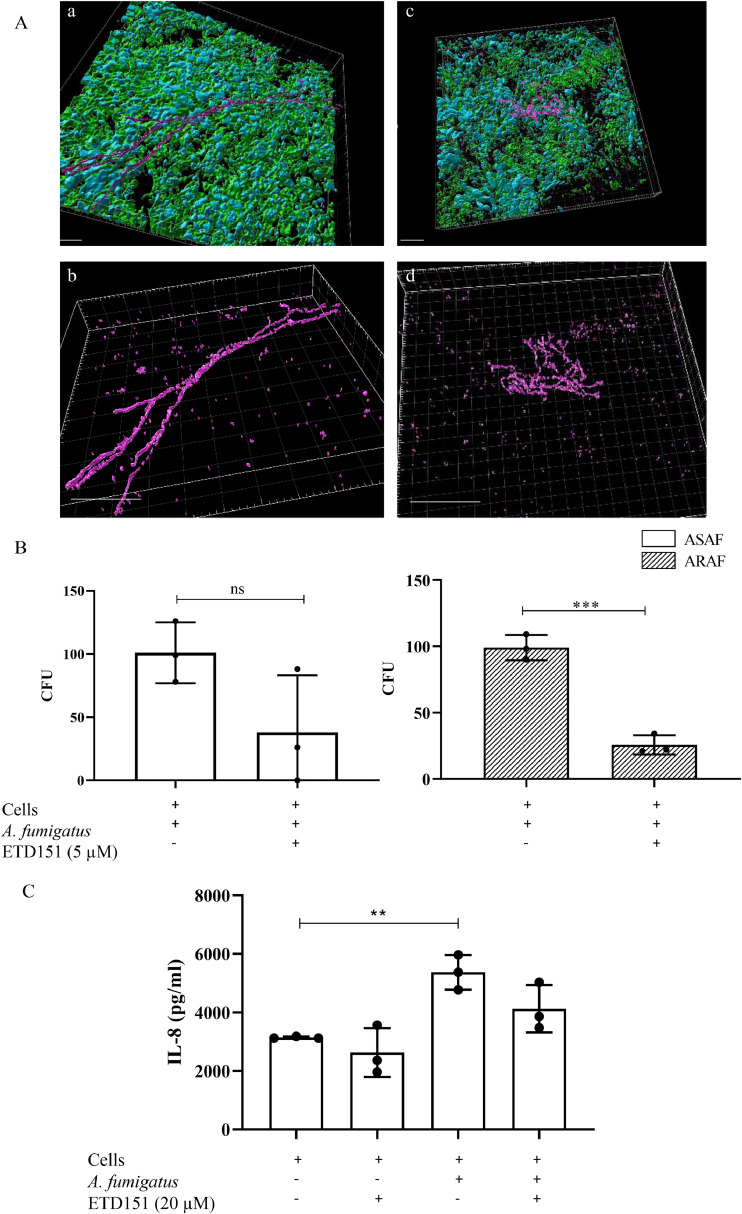
Antifungal activity of ETD151 against A. fumigatus in the PHBECs infection model. (A) Confocal immunofluorescence imaging of the GFP-expressing A. fumigatus ATCC13073 (magenta) infecting PHBECs (donor 1), stained with anti-claudin antibody (green), and anti-tubulin antibody (blue) to label cell junctions and cilia, respectively. Shown are representative images of untreated (a–b) and ETD151-treated (20 µM) (c– d) samples. Artificial colors were applied to enhance visualization. 3D reconstructions were performed using Imaris software. Scale bars: a and c = 30 µm; b and d = 20 µm. Images are representative of three independent experiments. (B) Quantification of fungal burden by CFU enumeration after 15 h of infection of PHBECs (donors 2 and 3) with ASAF (white bars) and ARAF (grey bars) strains, with or without 5 µM ETD151 treatment. Means of CFU per insert were calculated based on the results of three strains. Each point represents a technical simplicate. Statistical significance between CFU counts per insert in PHBECs infected and PHBECs infected and treated with ETD151 was determined using an unpaired ttest; ***p < 0.001. (C) IL-8 production by PHBECs (donor 1–3) after 15 hours of A. fumigatus infection. The means of IL-8 levels were calculated from results obtained with three tested strains. Each point represents a technical duplicate. Statistical significance between PHBECS infected with A. fumigatus and PHBECs unstimulated or PHBECs infected with A. fumigatus strains treated with ETD151 was determined using oneway ANOVA with Bonferroni’s multiple comparisons test. **p < 0.005.

To assess whether the morphological defects induced by ETD151 in cell-free cultures are maintained during epithelial infection, primary human bronchial epithelial cells (PHBECs) cultured at air-liquid interface (ALI) were infected with conidia of a GFP-expressing *A. fumigatus* strain (ATCC13073) for 15 h, with or without 20 µM of ETD151. Confocal microscopy revealed that untreated hyphae developed long, thin, poorly branched hyphae. In contrast, ETD151-treated hyphae appeared shortened and hyperbranched, consistent with observations from cell-free cultures (Fig. 9A).

To determine whether ETD151 can effectively reduce fungal growth in this model, fungal burden was quantified by measuring colony-forming units (CFU) following 15-hour treatment in infected PHBECs. ETD151 significantly reduced the CFU counts of ARAF strains by 73 % (±8 %) compared to untreated controls (p = 0.0006; *t*-test) (Fig. 9B). While a reduction was also observed in ASAF strains, the difference did not reach statistical significance (p = 0.1).

To evaluate the cytotoxicity of ETD151, we measured the transepithelial electrical resistance (TEER) of PHBECs infected or not with *A. fumigatus* and treated or not with ETD151 (5 µM or 20 µM). No significant difference in TEER was observed between ETD151-treated or untreated, and infected or uninfected PHBECs, indicating that ETD151 does not compromise epithelial barrier integrity under these conditions (Table S3).

We then analyze the potential immunomodulatory activity of ETD151 by measuring, in apical and basal compartments, IL-8 synthesis, a chemokine involved in the neutrophil’s recruitment. PHBEC were infected for 15 h with *A. fumigatus* in presence or not of ETD151. In the apical part, treatment of PHBEC with 20 µM ETD151 for 15 h did not significantly induce the secretion of IL-8, indicating no significant immunomodulatory effect of ETD151 by itself. The IL-8 synthesis induced by *A. fumigatus* that was not inhibited in presence of ETD151 (Fig. 9C). No modulation of IL-8 synthesis was observed in the basal compartment regardless of whether the PHBECs were infected or not, and whether ETD151 was added or not (data not shown). Importantly, IL-6 was not detected in either apical or basal compartments under any experimental condition (data not shown).

## 4 Discussion

The therapeutic landscape for *Aspergillus*-associated diseases remains critically limited, with only three classes of antifungals available (Wiederhold, 2018). Polyenes, azole derivatives, and echinocandins, have anti-*Aspergillus* activity but are associated with significant limitations due to their toxicity and/or pharmacokinetics. The context is even exacerbated with the emergence of ARAF strains that threaten the efficacy of current therapeutic regimens. In several geographic areas, including India, Iran, and the Netherlands, the prevalence of ARAF strains has prompted clinicians to replace azoles with amphotericin B for first-line therapy of invasive aspergillosis (Bosetti and Neofytos, 2023). In this context, AMPs have gained attention as potential antifungal candidates due to their overall favourable safety profile and the low risk of resistance emergence (Mihooliya and Kumari, 2024). Here, we investigated the antifungal activity of ETD151, a recombinant AMP, against both ASAF and ARAF clinical strains, and explored its mechanism of action, spectrum of effects, and implications for therapeutic use.

ETD151 did not prevent conidial germination but markedly altered hyphal development, the pathogenic form of *A. fumigatus*, causing hyperbranching and cytoplasmic leakage at hyphal tips. This phenotype resembles the morphological changes induced by echinocandins, which inhibit β−1,3-glucan synthase, resulting in apical stress and defective cell wall construction (Moreno-Velásquez et al., 2017).

However, our results, together with data in the literature, suggest a mechanism of action targeting the cytoplasmic membrane rather than the cell wall. Indeed, a *Δbar1* mutant lacking GlcCer, a key component of the membrane, revealed a similar hyperbranching phenotype and a marked resistance to ETD151. These findings strongly support a model in which GlcCer represents a primary target of ETD151, with disruption of the membrane lipid organization leading to cytoplasmic leakage and finally the inability of the fungus to grow. Moreover, recent biophysical data using microscale thermophoresis confirm that ETD151 specifically binds to GlcCer in *B. cinerea*, reinforcing the idea of a conserved lipid-based target across fungal species (Kharrat et al., 2025). Our hypothesis that GlcCer constitutes a primary target of ETD151 is further reinforced by competitive inhibition assays in which exogenous soy-derived GlcCer significantly reduced the antifungal activity of the peptide. However, supplementation with GlcCer did not restore metabolic activity in treated ARAF strains. This partial rescue highlights the complexity of membrane homeostasis and suggests that ETD151 may trigger downstream effects that are not reversible by membrane lipid repletion alone. On the other hand, GlcCer structure is conserved among numerous pathogenic fungi, including *Candida* spp., *Fusarium* spp., and *Cryptococcus* spp. Some of them are resistant to different classes of antifungal drugs, so future studies should assess the spectrum of ETD151 activity among pathogenic fungi beyond *A. fumigatus*. Positive results would bolster the clinical value of ETD151 as an important antifungal drug.

To gain further insight into the cellular pathways impacted by ETD151, we conducted a transcriptomic analysis of treated A. fumigatus hyphae. This approach aimed to identify broader physiological responses beyond the morphological and metabolic alterations observed. The data revealed significant modulation in the expression of genes involved in cell wall organization, membrane structure, and cell periphery processes. Among these, genes such as cat1, grg1, cspA, calA, nce102, and acuD were upregulated, whereas gta1 was downregulated. These changes suggest a coordinated stress response: upregulation of grg1 may reflect activation of general stress-protective mechanisms, while increased acuD expression indicates adjustments in carbon metabolism through the glyoxylate cycle (Suh et al., 2012; Flipphi et al., 2014). *Cat1* encodes a catalase involved in reactive oxygen species detoxification, suggesting activation of oxidative stress responses (Keizer et al., 2022). The downregulation of gta1, encoding a glutaminase, could affect nitrogen metabolism and protein turnover. Upregulation of nce102, involved in sporulation and membrane organization, alongside cspA and calA, which contribute to cell wall integrity, adhesion, and spore germination, suggests that the fungus attempts to reinforce its cell wall and stabilize membranes to counteract ETD151-induced disruption (Belaish et al., 2008; Khalaj et al., 2012; Levdansky et al., 2010; Liu et al., 2016). Collectively, these transcriptional patterns indicate that A. fumigatus responds to ETD151 by activating oxidative stress defenses, remodeling its cell wall, and reorganizing membranes, likely as compensatory mechanisms to maintain homeostasis and survive the antifungal insult.

The differential responses to ETD151 observed between ARAF and ASAF strains (hyphae permeabilization, impact of GlcCer supplementation and efficacy of antifungal drugs combination) highlight the multifactorial complexity of membrane-targeting antifungal strategies. Our permeabilization assays showed consistently that ARAF strains were more sensitive to ETD151 than ASAF strains, suggesting an increased vulnerability of their membrane architecture. Interestingly, while GlcCer supplementation was able to restore hyphal morphology in both strain types, but only ASAF strains recovered metabolic activity, pointing to a disconnect between structural and functional rescue in ARAF. This suggests that, in azole-resistant isolates, ETD151 may exert additional intracellular stress beyond GlcCer disruption. Given that azole resistance often involves changes in membrane sterol composition, lipid homeostasis, or stress response pathways, it is plausible that these adaptations render ARAF strains more susceptible to cumulative membrane disturbances and less capable of compensating through exogenous lipid supplementation (Khalaj et al. 2012; Liu et al. 2016; Belaish et al. 2008; Levdansky et al. 2010). Inefficiency of caspofungin and ETD151 combination on ARAF strains compared to ASAF strains supports a difference in their membrane composition underscoring the need for a comparative lipidomic analysis. Understanding potential differences in sphingolipid or sterol profiles between these clinical isolates could elucidate the molecular basis of their variable susceptibility. This could also help to identify biomarkers predictive of therapeutic response.

Unlike some AMPs for which acquired resistance has been documented, ETD151 selective pressure was not associated with the emergence of resistant strains. This contrasts with azoles, for which prolonged clinical exposure contributes to the emergence of azole-resistant strains. A useful comparison can be made with amphotericin B (AMB), a polyene that, like ETD151, targets fungal membrane components directly. AMB binds to ergosterol, a critical lipid in fungal membranes, disrupting membrane integrity and leading to cell death (Anderson et al., 2014). Acquired resistance to AMB remains rare, likely because ergosterol is essential for membrane structure and function (Lee et al., 2023; Cowen et al., 2014). Mutations in the ergosterol biosynthetic pathway (*e.g., ERG3, ERG6, ERG11*) typically incur a high fitness cost, resulting in decreased virulence and poor survival in murine models of invasive aspergillosis (Xie et al., 2024; Alcazar-Fuoli et al., 2006). Disruptions in GlcCer biosynthesis have been associated with major phenotypic defects, such as hyperbranching and loss of apical growth, as seen in Aspergillus. nidulans ∆*gcs*A mutant (Fernandes et al., 2016). Consistently, our A. fumigatus *∆bar1* mutant exhibited the same morphological alterations. Thus, resistance to ETD151 through GlcCer remodeling would likely impose a heavy physiological burden on fungal cells, as observed with the reduced diameter of *∆bar1* strains, and thereby may limit the likelihood of resistance emergence in natural populations.

Our study further demonstrated that ETD151 retains its antifungal activity in the presence of bronchial mucus produced by PHBECs. However, it is important to note that in the presence of epithelial cells, it is unclear whether ETD151 exerts a direct effect on A. fumigatus, as the cells may release other factors such as antimicrobial peptides, cytokines, or reactive oxygen species, which could contribute to fungal inhibition. In contrast, in our *in vitro* experiments performed in the absence of cells, the observed antifungal activity can be attributed entirely to ETD151.

When comparing the inhibition of A. fumigatus in presence or in absence of PHBECs, we observed similar effects of ETD151, such as morphological changes in the hyphae. However, a notable dissimilarity is that ETD151 appears more effective in the presence of cells. This observation supports the hypothesis that a synergistic antifungal effect may exist between ETD151 and the PHBEC, for which antifungal activity has already been described (Richard et al., 2018).

So, all these results support the idea that administration through inhalation could be applied efficiently to ETD151. Nebulization offers a non-invasive route of administration for obtaining high concentrations of drugs at the site of infection while minimizing systemic exposure and associated toxicity. This method is already in clinical use for delivering drugs such as colistin to CF patients (Bauldoff et al., 1997; Hodson et al., 2002). These properties strengthen the case for pursuing preclinical models of inhaled ETD151 therapy, for the treatment of *Aspergillus* bronchial or lung colonization.

Indeed, previous literature indicates that several factors within CF mucus, such as high viscosity, acidic pH, abundant proteases, and elevated concentrations of divalent cations (*e.g.*, Ca²⁺ and Mg²⁺), can reduce the efficacy of AMPs by impeding their diffusion, degrading their structure, or neutralizing their charge-based affinity for microbial membranes (Sala et al., 2021; Goldman et al., 1997; Taggart et al., 2003; Zhang et al., 2005; Hiemstra, 2007). To go further, it could be interesting to test its efficacy using bronchial cells obtained from CF patients.

In conclusion, ETD151 has a strong potential as a novel anti-*Aspergillus fumigatus* agent, with particular efficacy against azole-resistant *A. fumigatus* strains. Its retained activity in the presence of bronchial mucus, lack of toxicity on bronchial cells, and absence of observed resistance development make it an attractive candidate for nebulized therapy in patients with chronic forms of aspergillosis, such as bronchial colonisation or chronic pulmonary aspergillosis. Future work should focus on confirming it’s *in vivo* efficacy, defining its spectrum of activity, and optimizing its formulation for pulmonary delivery.

## Funding sources

CR received a doctoral fellowship from the French Ministry of Higher Education, Research, and Innovation. JG received grants from French cystic fibrosis non-profit organizations Vaincre la Mucoviscidose and Association Gregory Lemarchal (RF20230503260 and RF20240503525).

The Olympus FV3000 was obtained with financial support from ITMO Cancer of Aviesan within the 2021–2030 Cancer Control Strategy framework, on funds administered by Inserm.

## CRediT authorship contribution statement

**Camille Rochard:** Data curation, Formal analysis, Writing – original draft. **Jeanne Bigot:** Supervision, Writing – review & editing. **Nicolas Millet:** Writing – review & editing. **Viviane Balloy:** Methodology, Supervision, Writing – original draft. **Isabel Valsecchi:** Methodology, Writing – review & editing. **Françoise Botterel:** Writing – review & editing. **Romain Morichon:** Software. **Thierry Fontaine:** Formal analysis, Methodology, Writing – review & editing. **Loïc Guillot:** Writing – review & editing. **Philippe Bulet:** Writing – review & editing. **Christophe Hennequin:** Supervision, Writing – review & editing. **Juliette Guitard:** Conceptualization, Methodology, Project administration, Supervision, Writing – review & editing, Funding acquisition.

## Declaration of competing interest

The authors declare the following financial interests/personal relationships which may be considered as potential competing interests:

JULIETTE GUITARD reports financial support was provided by Vaincre la Mucoviscidose. Rochard Camille reports financial support was provided by Ministry of Higher Education and Research. The Olympus FV3000 was obtained with financial support from ITMO Cancer of Aviesan within the 2021–2030 Cancer Control Strategy framework, on funds administered by Inserm If there are other authors, they declare that they have no known competing financial interests or personal relationships that could have appeared to influence the work reported in this paper.

## Appendix Supplementary materials

Image, application 1

Image, application 2

## References

[bib0001] Abu-Farha R.K., Sobh S., Abu Hammour K., Darwish El-Hajji F., Shilbayeh S.A., Itani R. (2025). Prevalence, appropriateness, and outcomes of Colistin use in multidrug-resistant Pseudomonas aeruginosa infections: insights from Hospital data. Medicina.

[bib0003] Alcazar-Fuoli L., Mellado E., Garcia-Effron G., Buitrago M.J., Lopez J.F., Grimalt J.O., Cuenca-Estrella J.M., Rodriguez-Tudela J.L. (2006). Aspergillus fumigatus C-5 sterol desaturases Erg3A and Erg3B: role in sterol biosynthesis and antifungal drug susceptibility. Antimicrob. Agents Chemother..

[bib0004] Ames B.N. (1966). Methods in Enzymology, Complex Carbohydrates.

[bib0005] Anderson T.M., Clay M.C., Cioffi A.G., Diaz K.A., Hisao G.S., Tuttle M.D., Nieuwkoop A.J., Comellas G., Maryum N., Wang S., Uno B.E., Wildeman E.L., Gonen T., Rienstra C.M., Burke M.D. (2014). Amphotericin forms an extramembranous and fungicidal sterol sponge. Nat. Chem. Biol..

[bib0006] Aumer T., Voisin S.N., Knobloch T., Landon C., Bulet P. (2020). Impact of an antifungal insect defensin on the proteome of the phytopathogenic fungus botrytis cinerea. J. Proteome Res..

[bib0007] Bahar A.A., Ren D. (2013). Antimicrobial peptides. Pharm. Basel Switz..

[bib0008] Bauldoff G.S., Nunley D.R., Manzetti J.D., Dauber J.H., Keenan R.J. (1997). Use of aerosolized colistin sodium in cystic fibrosis patients awaiting lung transplantation. Transplantation.

[bib0009] Belaish R., Sharon H., Levdansky E., Greenstein S., Shadkchan Y., Osherov N. (2008). The Aspergillus nidulans cetA and calA genes are involved in conidial germination and cell wall morphogenesis. Fungal Genet. Biol. FG B.

[bib0010] Boparai J.K., Sharma P.K. (2020). Mini review on antimicrobial peptides, sources, mechanism and recent applications. Protein Pept. Lett..

[bib0011] Bosetti D., Neofytos D. (2023). Invasive aspergillosis and the impact of azole-resistance. Curr. Fungal Infect. Rep..

[bib0012] Browne K., Chakraborty S., Chen R., Willcox M.D., Black D.S., Walsh W.R., Kumar N. (2020). A new era of antibiotics: the clinical potential of antimicrobial peptides. Int. J. Mol. Sci..

[bib0013] Cassone M., Frith N., Vogiatzi P., Wade J.D., Otvos L. (2009). Induced resistance to the designer proline-rich antimicrobial peptide A3-APO does not involve changes in the intracellular target DnaK. Int. J. Pept. Res. Ther..

[bib0014] Cowen L.E., Sanglard D., Howard S.J., Rogers P.D., Perlin D.S. (2014). Mechanisms of antifungal drug resistance. Cold Spring Harb. Perspect. Med..

[bib0015] de Castro P.A., Pinzan C.F., Dos Reis T.F., Valero C., Van Rhijn N., Menegatti C., de Freitas Migliorini I.L., Bromley M., Trentin G., Almeida F., Fleming A.B., Traynor A.M., Sarikaya-Bayram Ö., Bayram O., Malavazi I., Ebel F., Barbosa J.C.J., Fill T., Pupo M.T., Goldman G.H. (2023). Vacuoles and peroxisomes are involved in Aspergillus fumigatus gliotoxin production and self-protection. Res. Sq..

[bib0016] Denning D.W. (2024). Global incidence and mortality of severe fungal disease. Lancet Infect. Dis..

[bib0017] Denning D.W. (2016). Minimizing fungal disease deaths will allow the UNAIDS target of reducing annual AIDS deaths below 500 000 by 2020 to be realized. Philos. Trans. R. Soc. Lond. B. Biol. Sci..

[bib0018] Desoubeaux G., Coste A.T., Imbert C., Hennequin C. (2022). Overview about Candida auris: what’s up 12 years after its first description?. J. Mycol. Medicale.

[bib0019] dos Reis T.F., de Castro P.A., Bastos R.W., Pinzan C.F., Souza P.F.N., Ackloo S., Hossain M.A., Drewry D.H., Alkhazraji S., Ibrahim A.S., Jo H., Lightfoot J.D., Adams E.M., Fuller K.K., deGrado W.F., Goldman G.H. (2023). A host defense peptide mimetic, brilacidin, potentiates caspofungin antifungal activity against human pathogenic fungi. Nat. Commun..

[bib0020] Fernandes C.M., de Castro P.A., Singh A., Fonseca F.L., Pereira M.D., Vila T.V.M., Atella G.C., Rozental S., Savoldi M., Del Poeta M., Goldman G.H., Kurtenbach E. (2016). Functional characterization of the spergillus nidulans glucosylceramide pathway reveals that LCB Δ8-desaturation and C9-methylation are relevant to filamentous growth, lipid raft localization and Psd1 defensin activity. Mol. Microbiol..

[bib0021] Fisher M.C., Denning D.W. (2023). The WHO fungal priority pathogens list as a game-changer. Nat. Rev. Microbiol..

[bib0022] Flipphi M., Oestreicher N., Nicolas V., Guitton A., Vélot C. (2014). The Aspergillus nidulans acuL gene encodes a mitochondrial carrier required for the utilization of carbon sources that are metabolized via the TCA cycle. Fungal Genet. Biol. FG B.

[bib0023] Goldman M.J., Anderson G.M., Stolzenberg E.D., Kari U.P., Zasloff M., Wilson J.M. (1997). Human beta-defensin-1 is a salt-sensitive antibiotic in lung that is inactivated in cystic fibrosis. Cell.

[bib0024] Gong J., Huang J., Liu Y., Zhang Y., Gao Y. (2024). Unveiling environmental transmission risks: comparative analysis of azole resistance in Aspergillus fumigatus clinical and environmental isolates from Yunnan. China. Microbiol. Spectr..

[bib0025] Hair P.I., Keam S.J. (2007). Daptomycin: a review of its use in the management of complicated skin and soft-tissue infections and Staphylococcus aureus bacteraemia. Drugs.

[bib0026] Hancock R.E. (2001). Cationic peptides: effectors in innate immunity and novel antimicrobials. Lancet Infect. Dis..

[bib0027] Haney E.F., Mansour S.C., Hancock R.E.W. (2017). Antimicrobial peptides: an introduction. Methods Mol. Biol. Clifton NJ.

[bib0028] He X., Kusuya Y., Hagiwara D., Toyotome T., Arai T., Bian C., Nagayama M., Shibata S., Watanabe A., Takahashi H. (2024). Genomic diversity of the pathogenic fungus Aspergillus fumigatus in Japan reveals the complex genomic basis of azole resistance. Commun. Biol..

[bib0029] Helmerhorst E.J., Troxler R.F., Oppenheim F.G. (2001). The human salivary peptide histatin 5 exerts its antifungal activity through the formation of reactive oxygen species. Proc. Natl. Acad. Sci. U. S. A..

[bib0030] Hiemstra P.S. (2007). Antimicrobial peptides in the real world: implications for cystic fibrosis. Eur. Respir. J..

[bib0031] Hodson M.E., Gallagher C.G., Govan J.R.W. (2002). A randomised clinical trial of nebulised tobramycin or colistin in cystic fibrosis. Eur. Respir. J..

[bib0032] Hong R., Xie A., Jiang C., Guo Y., Zhang Y., Chen J., Shen X., Li M., Yue X. (2024). A review of the biological activities of lactoferrin: mechanisms and potential applications. Food Funct..

[bib0033] Jabet A., Normand A.-C., Brun S., Dannaoui E., Bachmeyer C., Piarroux R., Hennequin C., Moreno-Sabater A. (2023). Trichophyton indotineae, from epidemiology to therapeutic. J. Mycol. Medicale.

[bib0034] Keizer E., Valdes I., McCann B., Bignell E., Wösten H., de Cock H. (2022). The protective role of 1,8-dihydroxynaphthalene–Melanin on conidia of the opportunistic Human pathogen Aspergillus fumigatus revisited: no role in protection against hydrogen peroxide and superoxides. mSphere.

[bib0035] Khalaj V., Azizi M., Enayati S., Khorasanizadeh D., Ardakani E.M. (2012). NCE102 homologue in Aspergillus fumigatus is required for normal sporulation, not hyphal growth or pathogenesis. FEMS Microbiol. Lett..

[bib0036] Kharrat O., Yamaryo-Botté Y., Nasreddine R., Voisin S., Aumer T., Cammue B.P.A., Madinier J.-B., Knobloch T., Thevissen K., Nehmé R., Aucagne V., Botté C., Bulet P., Landon C. (2025). The antimicrobial activity of ETD151 defensin is dictated by the presence of glycosphingolipids in the targeted organisms. Proc. Natl. Acad. Sci. U. S. A..

[bib0037] Kim, H.J., 2014. Exploitation of reactive oxygen species by fungi: roles in host-fungus interaction and fungal development 24, 1455–1463. 10.4014/jmb.1407.07072. 25152060

[bib0038] Krupińska A.M., Bogucki Z. (2024). Lactoferrin as a potential therapeutic for the treatment of Candida-associated denture stomatitis. J. Oral Biosci..

[bib0039] Larwood D.J. (2020). Nikkomycin Z—Ready to meet the promise?. J. Fungi.

[bib0040] Latgé J.-P., Chamilos G. (2019). Aspergillus fumigatus and aspergillosis in 2019. Clin. Microbiol. Rev..

[bib0041] Lee J.-K., Park S., Kim Y.-M., Guk T., Choi J.K., Kim J.-Y., Lee M.-Y., Jang M.-K., Park S.-C. (2022). Antifungal and anti-inflammatory activities of PS1-2 peptide against fluconazole-resistant Candida albicans. Antibiot. Basel Switz..

[bib0042] Lee Y., Robbins N., Cowen L.E. (2023). Molecular mechanisms governing antifungal drug resistance. Npj Antimicrob. Resist..

[bib0043] Levdansky E., Kashi O., Sharon H., Shadkchan Y., Osherov N. (2010). The Aspergillus fumigatus cspA gene encoding a repeat-rich cell wall protein is important for normal conidial cell wall architecture and interaction with host cells. Eukaryot. Cell.

[bib0044] Liu H., Lee M.J., Solis N.V., Phan Q.T., Swidergall M., Ralph B., Ibrahim A.S., Sheppard D.C., Filler S.G. (2016). Aspergillus fumigatus CalA binds to integrin α5β1 and mediates host cell invasion. Nat. Microbiol..

[bib0045] Lockhart S.R., Frade J.P., Etienne K.A., Pfaller M.A., Diekema D.J., Balajee S.A. (2011). Azole resistance in Aspergillus fumigatus isolates from the ARTEMIS global surveillance study is primarily due to the TR/L98H mutation in the cyp51A gene. Antimicrob. Agents Chemother..

[bib0046] McHugh J., Chesdachai S., Dunsirn M., Wengenack N., Vergidis P. (2025). Increasing fluconazole resistance in Candida parapsilosis: a 10-year analysis of blood culture isolates at a US reference laboratory (2015–2024). J. Infect. Dis..

[bib0047] Mellado E., Diaz-Guerra T.M., Cuenca-Estrella M., Rodriguez-Tudela J.L. (2001). Identification of two different 14-alpha sterol demethylase-related genes (cyp51A and cyp51B) in Aspergillus fumigatus and other Aspergillus species. J. Clin. Microbiol..

[bib0048] Mihooliya K.N., Kumari A., Baindara P., Mandal S.M. (2024). Evolution of Antimicrobial Peptides: From Self-Defense to Therapeutic Applications.

[bib0049] Moreno-Velásquez S.D., Seidel C., Juvvadi P.R., Steinbach W.J., Read N.D. (2017). Caspofungin-mediated growth inhibition and paradoxical growth in Aspergillus fumigatus involve fungicidal hyphal tip lysis coupled with regenerative intrahyphal growth and dynamic changes in β-1,3-glucan synthase localization. Antimicrob. Agents Chemother..

[bib0050] Morio F., Aubin G.G., Danner-Boucher I., Haloun A., Sacchetto E., Garcia-Hermoso D., Bretagne S., Miegeville M., Le Pape P. (2012). High prevalence of triazole resistance in Aspergillus fumigatus, especially mediated by TR/L98H, in a French cohort of patients with cystic fibrosis. J. Antimicrob. Chemother..

[bib0051] Patterson T.F., Thompson G.R., Denning D.W., Fishman J.A., Hadley S., Herbrecht R., Kontoyiannis D.P., Marr K.A., Morrison V.A., Nguyen M.H., Segal B.H., Steinbach W.J., Stevens D.A., Walsh T.J., Wingard J.R., Young J.-A.H., Bennett J.E. (2016). Practice Guidelines for the Diagnosis and Management of Aspergillosis: 2016 update by the Infectious Diseases Society of America. Clin. Infect. Dis. Off. Publ. Infect. Dis. Soc. Am..

[bib0052] Richard N., Marti L., Varrot A., Guillot L., Guitard J., Hennequin C., Imberty A., Corvol H., Chignard M., Balloy V. (2018). Human bronchial epithelial cells inhibit Aspergillus fumigatus germination of extracellular conidia via FleA recognition. Sci. Rep..

[bib0053] Rima Mariam, Rima Mohamad, Fajloun Z., Sabatier J.-M., Bechinger B., Naas T. (2021). Antimicrobial peptides: a potent alternative to antibiotics. Antibiot. Basel Switz..

[bib0054] Sala V., Cnudde S.J., Murabito A., Massarotti A., Hirsch E., Ghigo A. (2021). Therapeutic peptides for the treatment of cystic fibrosis: challenges and perspectives. Eur. J. Med. Chem..

[bib0055] Scott M.G., Davidson D.J., Gold M.R., Bowdish D., Hancock R.E.W. (2002). The human antimicrobial peptide LL-37 is a multifunctional modulator of innate immune responses. J. Immunol. Baltim. Md.

[bib0056] Singh A., Sharma B., Mahto K.K., Meis J.F., Chowdhary A. (2020). High-frequency direct detection of triazole resistance in Aspergillus fumigatus from patients with chronic pulmonary fungal diseases in India. J. Fungi Basel Switz..

[bib0057] Siopi M., Georgiou P.-C., Pournaras S., Meletiadis J. (2023). Optimization of the EUCAST reference broth microdilution method for echinocandin susceptibility testing of Aspergillus fumigatus. J. Antimicrob. Chemother..

[bib0058] Snelders E., van der Lee H.A.L., Kuijpers J., Rijs A.J.M.M., Varga J., Samson R.A., Mellado E., Donders A.R.T., Melchers W.J.G., Verweij P.E. (2008). Emergence of azole resistance in Aspergillus fumigatus and spread of a single resistance mechanism. PLoS Med..

[bib0059] Suh M.-J., Fedorova N.D., Cagas S.E., Hastings S., Fleischmann R.D., Peterson S.N., Perlin D.S., Nierman W.C., Pieper R., Momany M. (2012). Development stage-specific proteomic profiling uncovers small, lineage specific proteins most abundant in the Aspergillus Fumigatus conidial proteome. Proteome Sci..

[bib0060] Taggart C.C., Greene C.M., Smith S.G., Levine R.L., McCray P.B., O’Neill S., McElvaney N.G. (2003). Inactivation of human beta-defensins 2 and 3 by elastolytic cathepsins. J. Immunol. Baltim. Md.

[bib0061] Takeda K., Suzuki J., Watanabe A., Sekiguchi R., Sano T., Watanabe M., Narumoto O., Kawashima M., Fukami T., Sasaki Y., Tamura A., Nagai H., Matsui H., Kamei K. (2022). The accuracy and clinical impact of the morphological identification of Aspergillus species in the age of cryptic species: a single-centre study. Mycoses.

[bib0062] Thevissen K., Warnecke D.C., François I.E.J.A., Leipelt M., Heinz E., Ott C., Zähringer U., Thomma B.P.H.J., Ferket K.K.A., Cammue B.P.A. (2004). Defensins from insects and plants interact with fungal glucosylceramides. J. Biol. Chem..

[bib0063] van Rhijn N., Arikan-Akdagli S., Beardsley J., Bongomin F., Chakrabarti A., Chen S.C.-A., Chiller T., Lopes Colombo A., Govender N.P., Alastruey-Izquierdo A., Kidd S.E., Lackner M., Li R., Hagen F. (2024). Beyond bacteria: the growing threat of antifungal resistance. Lancet Lond. Engl..

[bib0064] Wiederhold N.P. (2018). The antifungal arsenal: alternative drugs and future targets. Int. J. Antimicrob. Agents.

[bib0065] Wiederhold N.P., Patterson T.F. (2015). Emergence of Azole resistance in Aspergillus. Semin. Respir. Crit. Care Med..

[bib0066] Xie J., Rybak J.M., Martin-Vicente A., Guruceaga X., Thorn H.I., Nywening A.V., Ge W., Parker J.E., Kelly S.L., Rogers P.D., Fortwendel J.R. (2024). The sterol C-24 methyltransferase encoding gene, erg6, is essential for viability of Aspergillus species. Nat. Commun..

[bib0067] Zhang L., Parente J., Harris S.M., Woods D.E., Hancock R.E.W., Falla T.J. (2005). Antimicrobial peptide therapeutics for cystic fibrosis. Antimicrob. Agents Chemother..

